# Role and Diagnostic Significance of Apolipoprotein D in Selected Neurodegenerative Disorders

**DOI:** 10.3390/diagnostics14242814

**Published:** 2024-12-14

**Authors:** Agata Kolanek, Roman Cemaga, Mateusz Maciejczyk

**Affiliations:** 1 Students’ Scientific Club “Biochemistry of Civilization Diseases” at the Department of Hygiene, Epidemiology and Ergonomics, Medical University of Bialystok, 15-233 Bialystok, Poland; agkolanek@gmail.com (A.K.); rcemaga@gmail.com (R.C.); 2 Department of Hygiene, Epidemiology and Ergonomics, Medical University of Bialystok, 2c Mickiewicza Street, 15-233 Bialystok, Poland

**Keywords:** apolipoprotein D, biomarker, central nervous system, multiple sclerosis, Alzheimer’s disease, Parkinson’s disease, apolipoproteins, neurological diseases, neurodegenerative disorders

## Abstract

The World Health Organization in 2021 ranked Alzheimer’s disease and other dementias as the seventh leading cause of death globally. Neurodegenerative disorders are progressive, intractable, and often fatal diseases. Early diagnosis may allow patients to enjoy prolonged survival with attenuated symptomatology because of early intervention. Hence, further research on finding non-invasive biomarkers of neurodegenerative diseases is warranted. Apolipoprotein D (ApoD) is a glycoprotein involved in lipid metabolism, oxidative stress regulation, and inflammation. It is expressed in various body fluids and regions of the central nervous system. ApoD’s roles in neuroprotection, lipid transport, and anti-inflammatory processes are crucial as far as the prevention of neurodegenerative pathologies is concerned. This review aims to summarize the background knowledge on ApoD, and it covers studies indexed in the PubMed, Scopus, and Web of Science databases. It discusses the evidence for the multifaceted roles of ApoD in the mechanisms and pathogenesis of multiple sclerosis, Alzheimer’s disease, and Parkinson’s disease. ApoD may be a specific, sensitive, easily obtained, cost-effective biomarker for neurodegenerative diseases and its applications in diagnostic practices, treatment strategies, and advancing neurodegenerative disorders’ management.

## 1. Introduction

Neurodegenerative disorders (NDs) constitute a diverse group of pathologies of the brain and spinal cord, including diseases such as multiple sclerosis (MS), Alzheimer’s disease (AD), and Parkinson’s disease (PD). NDs are the second leading cause of death globally, accounting for 9 million deaths a year [1]. NDs may have common causative factors that include stress, drugs, traumatic events, or genetic background. Various molecular mechanisms are said to be involved in the pathogenesis of central nervous system (CNS) diseases. Those include alterations in lipid metabolism, oxidative stress, and inflammation [2,3,4]. Such diversity at the molecular level explains the variability of symptoms in CNS diseases [1,5,6]. Treatment of NDs is usually aimed at delaying disease progression rather than achieving a successful cure; thus, most NDs progress over time. Consequently, NDs are a significant cause of disability globally, contributing substantially to healthcare costs and economic burden [1].

Current biomarkers of NDs have significant limitations. Magnetic resonance imaging (MRI) is expensive, not widely available (especially in low-income countries), and not adequately specific, as the atrophic changes seen in AD can also occur due to aging or other NDs [7]. Similarly, cerebrospinal fluid (CSF) biomarkers, including tau protein [8], amyloid-β [9], serum neurofilament light chain (sNfL) [10], and α-synuclein [11], show insufficient specificity in distinguishing between NDs. In addition, classical ND biomarkers require invasive lumbar puncture and have limitations in terms of tracking disease progression. This is why finding an easily obtained biomarker may be essential for predicting, diagnosing, and monitoring medical response to NDs [12,13].

Apolipoproteins (Apo) are serum proteins that form the protein components of lipoprotein particles, notably high-density lipoproteins (HDLs) and low-density lipoproteins (LDLs) [14]. They are vital for regulating various biological processes, including lipid transport and metabolism [15], modulation of inflammatory responses [16,17], and protection against oxidative stress [18,19,20]. Studies demonstrate the use of Apo in the screening of cardiovascular disease [21], cancers [22], liver diseases [23], and diabetes [24]. Since cholesterol is vital for cerebral development, there has been an increasing interest in examining Apo’s role in NDs [25]. Numerous studies have revealed the involvement of apolipoprotein A (ApoA), apolipoprotein B (ApoB), apolipoprotein C (ApoC), apolipoprotein D (ApoD), apolipoprotein E (ApoE), apolipoprotein H (ApoH), and apolipoprotein J (ApoJ) in ND pathogenesis [26]. ApoD has garnered significant interest in ND pathology [25].

Apolipoprotein D, also called a progesterone-binding cyst protein (PBCP) or a gross cystic disease fluid protein 24 (GCDFP-24) [27], is a small glycoprotein first isolated from HDLs in 1973 by McConathy and Alaupovic [28]. It does not share amino acid homologies with other apolipoproteins and is molecularly similar to the lipocalin family of proteins [29,30]. The *ApoD* gene is located on human chromosome 3 [31]. The ApoD promoter contains various regulatory elements for steroids, fatty acids, estrogen, and components involved in the immune response, such as nuclear factor kappa B (NF-kB). That number of regulatory elements contributes to the complex regulation of ApoD expression [3,32,33,34].

ApoD is expressed in various organs and tissues such as the liver [35,36], intestine [35,36], pancreas [35,36], kidney [35,36], placenta [35], spleen [37], adrenal gland [37], and the CNS, mainly in a white matter [37], subarachnoid space area [36,37], dorsolateral prefrontal cortex [38,39], occipital cortex [40], substantia nigra [41], hippocampus [41], and cerebellum [4,37,39]. ApoD is expressed primarily by the glial cells (astrocytes, oligodendrocytes). ApoD has been found at the cellular level in the rough endoplasmic reticulum (RER), Golgi apparatus, lysosomes, endosomes, the outer side of the plasma membrane, and multivesicular bodies. However, it has not been found in mitochondria, peroxisomes, and cellular nuclei [27]. ApoD is also present in the body fluids, including plasma [28], cerebrospinal fluid [42,43], perilymph [43], urine [44], and secretions from exocrine glands (sweat [45], tears [46], and mammary secretions [27,47]).

The crystal structure of ApoD has revealed an eight-stranded antiparallel β-barrel flanked by α-helix that is typical for the lipocalin family [48]. ApoD contains an N-terminal signal peptide, allowing ApoD to enter the endoplasmic reticulum and undergo further modification during glycosylation [27]. Glycosylation allows ApoD to acquire new biological properties. Nevertheless, some studies suggest that ApoD glycosylation patterns may also be specific for CNS diseases. Qin et al. have shown an increase in ApoD α2–3 sialoglycolysation in children with autism spectrum disorder [49]. Therefore, exploring new glycosylation patterns of ApoD could provide useful diagnostic tools in the future. Glycosylation is also responsible for the different molecular weights of ApoD in tissues. Human brain ApoD has a lower molecular weight (29 kDa) than the tissue and plasma (32 kDa). Given that plasma does not physiologically contain the 29 kDa form of ApoD, ApoD can be used to predict blood–brain barrier disorders occurring in CNS diseases [25,50].

Due to the prevalence of ApoD in various body fluids, those fluids have the potential to serve as valuable diagnostic indicators. For example, detecting the ApoD protein or mRNA in the circulating fluids will likely be used to identify and track neuropathological conditions [25]. Several studies suggest that ApoD levels vary with the progression of NDs [51,52,53]. Detecting ApoD in the early phases of a disease or monitoring its changes over time holds significant potential for diagnosing NDs [54]. Additionally, ApoD can be measured in less invasive samples such as plasma, urine, and exocrine secretions, which reduces patient anxiety and simplifies the screening process [16]. Early detection of ApoD may facilitate intervention, mitigate symptoms, improve quality of life and longevity, and reduce healthcare costs for patients with NDs.

There has been a lot of interest in the role of ApoD in recent years, and several new clinical trials have been published regarding its involvement in NDs [40,55,56,57]. To date, no review has clarified the role of ApoD in NDs. In this study, we have reviewed the literature focused on ApoD, its role in pathophysiology, and its diagnostic significance in AD, MS, and PD.

## 2. Biological Functions of Apolipoprotein D

ApoD plays multiple roles in the body, as it can bind to various ligands, including progesterone, arachidonic acid (AA), retinoic acid, and pregnenolone [50]. Such a variety of ligands suggests that ApoD is involved in the modulation of various functions across the body. ApoD is involved in inflammatory response, lipid metabolism, oxidative stress regulation, synaptic reorganization, and reinnervation.

### 2.1. ApoD and Neuronal Function

**Figure 1:**
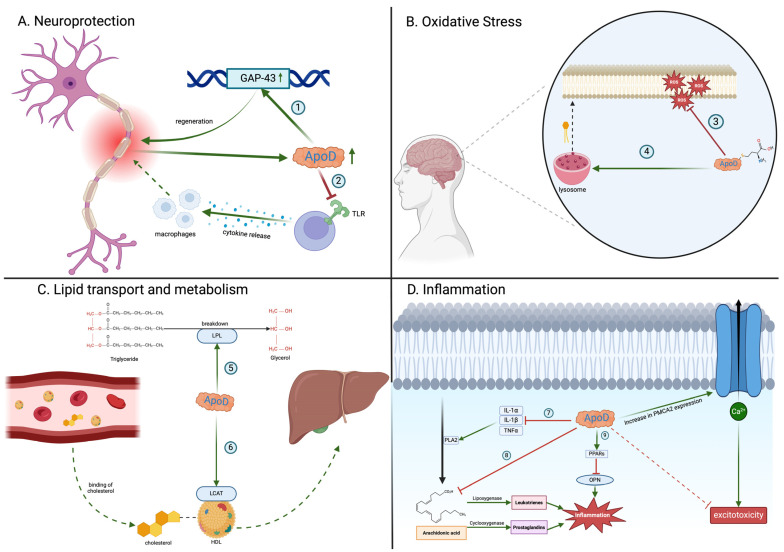
Biological functions of apolipoprotein D (ApoD). ApoD participates in (1) activation of *GAP-43*, a gene that promotes axonal regeneration; (2) inhibition of Toll-like receptors (TLRs), which mitigates excessive inflammation and macrophage infiltration at the injury site; (3) reduction in lipid peroxides through the methionine 93 residue on ApoD; (4) preservation of phospholipid membrane integrity by modulating lysosomal membrane stability; (5) activation of lipoprotein lipase (LPL), which assists in the breakdown of triglycerides into smaller molecules; (6) activation of the lecithin–cholesterol acyltransferase (LCAT) enzyme, which is responsible for esterifying cholesterol on HDLs and facilitating its transport to the liver; (7) inhibition of proinflammatory cytokines that activate phospholipase 2 (PLA2), responsible for liberating arachidonic acid from the phospholipid membrane; (8) direct binding of arachidonic acid, preventing its conversion into proinflammatory molecules; (9) activation of peroxisome proliferator-activated receptor (PPAR), resulting in the inhibition of osteopontin (OPN), a proinflammatory agent. ApoD: apolipoprotein D; GAP-43: growth-associated protein; HDL: high-density lipoprotein; IL-1α: interleukin-1α; IL-1β: interleukin-1β; LCAT: lecithin–cholesterol acyltransferase; LPL: lipoprotein lipase; OPN: osteopontin; PLA2: phospholipase 2; PMCA2: plasma membrane calcium ATPase type 2; PPARs: peroxisome proliferator-activated receptors; ROS: reactive oxygen species; TLRs: Toll-like receptors; TNFα; tumor necrosis factor-α. Created with https://BioRender.com.

ApoD has a variety of functions in the CNS (Figure 1A). Ganfornina et al. [58] have proved that ApoD regulates neurons’ myelin sheath thickness and impulse conduction. They demonstrated that the ApoD knockout (ApoD-KO) mice had elevated levels of the *Pmp22* gene, which is associated with neuronal demyelination [58,59]. Furthermore, the ApoD-KO mice showed impaired axonal regeneration, remyelination, and more significant functional impairment after injury compared to the wild-type mice, suggesting that ApoD is essential for nerve regeneration [58]. ApoD helps clear out myelin by Schwann cells (SC) and macrophages at the injury site, as it contains molecules that inhibit axonal regeneration. Ganfornina et al. [58] also hypothesized that ApoD might activate genes that aided in axonal regeneration, such as growth-associated protein 43 (GAP-43) [58]. Several other studies have confirmed that theory by noting an increase in ApoD in the injury site [60,61]. Garcia-Mateo et al. [62] noticed that ApoD worked as a ‘break’ to inflammation by hindering Toll-like receptors (TLRs). It was shown that without ApoD, the macrophage recruitment was accelerated, thereby prolonging the inflammatory process [62]. These findings are especially relevant to NDs, where impaired neuronal repair can exacerbate disease progression [63].

### 2.2. Apolipoprotein D and Oxidative Stress

ApoD also manages oxidative stress (Figure 1A). Oxidative stress is the imbalance between reactive oxidative species (ROS) and antioxidants, leading to disturbances in cellular metabolism via oxidation [20,64]. Since the brain produces most of the ATP in the body and the mitochondria constitute the primary source of ROS, redox imbalance is particularly potent there. The brain also contains significant amounts of lipids, which are particularly susceptible to oxidative damage. The production of lipid peroxides (e.g., lipid hydroperoxides (LOOHs)) may lead to membrane disruptions and the creation of toxic intermediates, e.g., malondialdehyde (MDA) and 4-hydroxynonenal (4-HNE) [65]. ApoD has been found to act as a homeostatic mechanism to counter oxidative stress in the brain. Experimental models demonstrate that the absence of ApoD leads to premature brain aging, neuronal loss, cognitive decline, and increased susceptibility to neurodegenerative disorders [66]. The antioxidant activity of ApoD may be due to its structural properties. ApoD contains methionine residues (ApoD-Met93), which are particularly susceptible to oxidation [67]. After exposure to LOOHs, the Met93 residue reduces LOOHs to less reactive lipid hydroperoxides (LOHs) and converts them to methionine sulfoxide (ApoD-MetSO): ApoD-Met93 + LOOHs → ApoD-MetSO + LOHs [20,68]. The oxidized form of ApoD (ApoD-Met-SO) is biologically unstable and capable of forming homodimers. Consequently, the dimeric form of ApoD may be detected in oxidative stress-mediated illnesses such as AD [69]. To maintain the antioxidant properties, ApoD-Met-SO must be reduced using the methionine sulfoxide reductase (MRS) [27]. Therefore, the ApoD antioxidant capacity is limited by MRS activity. Furthermore, it was suggested that ApoD indirectly modulates oxidative stress by changing the phospholipid membrane’s composition. ApoD affects the integrity of lysosomal membranes. Consequently, ApoD alterations might compromise cell membrane integrity, making it more vulnerable to oxidative stress [27].

The role of ApoD in managing oxidative stress has further been confirmed in in vivo and in vitro studies. For example, mice with impaired ApoD function exhibited elevated peroxide lipid levels in their brains, increasing their susceptibility to oxidative stress. Conversely, mice with upregulated ApoD levels showed a reduction in lipid peroxides [70]. Pascua-Maestro et al. (2019) showed that astrocytes were involved in ApoD paracrine secretion through the extracellular vesicles (EVs). Neurons that obtained ApoD showed better viability under paraquat (PQ)-induced oxidative stress than those that did not receive ApoD. This study has also revealed that neurons absorbed ApoD only in the EVs and not in the free-soluble form [71]. That suggests that the systemic administration of ApoD in the form of exosomes could be a potential treatment for neurodegenerative diseases.

ApoD’s antioxidant effects may have particular clinical implications in NDs. By diminishing ROS production and stabilizing membranes, ApoD can preserve appropriate neuronal function [3,4,72]. Fluctuations in ApoD levels may represent an active pathological process and suggest a new therapeutic target [69,72,73].

### 2.3. Apolipoprotein D and Lipid Regulation

ApoD, similarly to other apolipoproteins, is also involved in regulating lipid transport and metabolism (Figure 1D) [73]. Patients with lipid elevations exhibit a rise in ApoD, indicating that ApoD is required for the cleavage of cholesterol in peripheral tissue. Since ApoD possesses minimal binding activity to cholesterol, its role in cholesterol metabolism is linked to its interaction with the lecithin–cholesterol acyltransferase (LCAT) that esterifies free cholesterol on HDL particles and ultimately facilitates its transport to the liver [73]. ApoD is also implicated in activating lipoprotein lipase (LPL), the enzyme that promotes the breakdown of triglycerides [74]. ApoD insufficiency is correlated with a 35% reduction in LPL levels in adipose tissue and an increase in triglyceride levels in both young and old mice [75]. Studies on ApoE knockout mice (with induced atherosclerosis) have also revealed a significant elevation in ApoD plasma levels compared to the control group [30]. Moreover, a survey of 722 African people from Nigeria discovered a correlation between polymorphisms and mutations in the *ApoD* gene and plasma lipid levels. For example, the polymorphism at codon 36 (Phe366Val) was associated with decreased high-density lipoprotein cholesterol subtype 3 (HDL3-C) levels. In contrast, the polymorphism at codon 158 (Thr158Lys) was linked to higher levels of lipoprotein(a) Lp(a) in females and triglycerides in males [76]. This explains why diseases associated with lipid impairment, such as coronary artery disorders (CADs), show an increase in ApoD levels in their HDLs, correlating with ApoD levels [77]. It is essential to evaluate the role of ApoD in the brain tissue as the brain contains a significant concentration of lipids. Their modifications have been linked to many NDs. Higher concentrations of ApoD have been detected in the brain’s white matter and peri-infarct regions after a stroke, suggesting that ApoD plays a role in transporting essential molecules such as cholesterol and phospholipids. Those molecules are required for myelin formation, remodeling, and regenerating brain damage [78]. Altered ApoD levels may provide benefits in diagnosing lipid dysregulation associated with NDs.

### 2.4. Apolipoprotein D and Inflammation

ApoD also regulates immune responses and inflammation (Figure 1C). While controlled inflammation helps eliminate damaging agents and is involved in neuronal regeneration, chronic and uncontrolled inflammation is harmful and unwanted. Several studies highlight the anti-inflammatory properties of ApoD [20,79,80]. ApoD overexpression in mice has decreased plasma interleukin-6 (IL-6) and tumor necrosis factor-α (TNF-α) levels [80]. This, in turn, reduces the overabundant infiltration of T cells, which causes protective effects on the brain [79]. ApoD is also believed to activate peroxisome proliferator-activated receptors (PPARs), suppressing osteopontin, another well-known proinflammatory agent [80]. Research conducted on an animal model of coronavirus-induced encephalitis (HCoV-OC43) has demonstrated that mice with elevated levels of ApoD have restricted the activity of phospholipase 2 (PLA2), an enzyme involved in liberating AA from the phospholipid membrane. Consequently, mice with greater ApoD displayed improved viability due to inflammation suppression. The action of ApoD on PLA2 can be associated with reduced cytokine production, including interleukin-1α (IL-1α), interleukin-1β (IL-1β), and TNF-α [79]. ApoD can be essential in inflammation because AA is one of the main precursors for other proinflammatory agents [81]. ApoD has also been found to bind free AA in the cytosol, preventing its action in the inflammatory process [79,82]. ApoD overexpression may indirectly lead to a decrease in microglial activation. This process involves sequestering AA, which limits the activity of cyclooxygenase-2 (COX-2), a critical proinflammatory enzyme in the AA cascade [83]. Moreover, it was found that ApoD is assimilated by the microglial cells, where it reduces the release of IL-6, IL-10, and TNF-α in response to inflammation triggered by amyloid-β [84]. This evidence suggests that ApoD directly influences intracellular crosstalk in microglia. However, the precise mechanism of this action is still unclear [85].

ApoD also modulates excitotoxicity, a process associated with NDs that leads to neuronal death by the excessive activation of glutamatergic receptors [83]. The excitotoxicity results from an abnormal influx of calcium ions into the cell. ApoD was observed to enhance the expression of plasma membrane calcium ATPase type 2 (PMCA2), thereby facilitating Ca^2+^ efflux and buffering excess Ca^2+^. In the same study, ApoD was also discovered to reduce the amounts of the N-methyl-D-aspartate receptor (NMDAR) 2B subunit (NR2B), a factor associated with increased neuronal apoptosis [83].

ApoD regulates pathological processes associated with developing NDs, such as diminishing oxidative stress (Figure 1B), modulating lipid transport and inflammation, and regenerating neurons after injury. Since chronic inflammation and immune cell infiltration are hallmarks of NDs [86], ApoD, with its anti-inflammatory functions, could be explored as a potential biomarker able to address the inflammatory state of the brain. Moreover, the fact that ApoD can modulate the immune response could open up new therapeutic possibilities for treating NDs.

## 3. The Role of ApoD in Neurodegenerative Disorders

In the CNS, ApoD is expressed in white matter, the subarachnoid space area, dorsolateral prefrontal cortex, occipital cortex, substantia nigra, hippocampus, and cerebellum [4,36,37,38,39,40]. Glial cells (astrocytes, macrophages, and oligodendrocytes) also expressed ApoD protein [2]. ApoD is crucial in metabolizing plasma lipoproteins, lipid transport, and phospholipid metabolism in the brain [4,72]. ApoD also prevents neuronal oxidative damage, a common factor in NDs, and exhibits anti-inflammatory effects by reducing microglial activation [20,66]. In more detail, ApoD plays a neuroprotective role by repairing damaged lipid membranes, modulating astrocyte reactivity, and eliminating neurotoxic molecules discharged during cell death [54,87]. ApoD may, therefore, contribute to reinnervation, axonal regeneration, and synaptic reorganization [58,59]. ApoD may also constitute a therapeutic/diagnostic target in NDs. ApoD is present in CSF and blood and may be used in ND diagnostics.

### 3.1. Parkinson’s Disease

**Figure 2:**
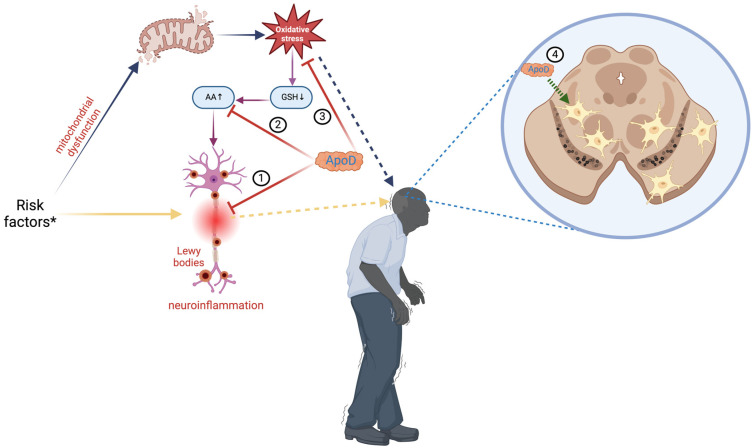
Role of apolipoprotein D in Parkinson’s disease. Multiple risk factors * (age, genetics, medications, environmental triggers, trauma) contribute to the etiology of Parkinson’s disease (PD). They can result in neuroinflammation, which causes the deposition of Lewy bodies involved in neuronal disruptions (yellow arrows), or disrupt mitochondrial activity, resulting in excessive oxidative stress (blue arrows). Oxidative stress might cause a reduction in glutathione (GSH) levels, leading to an elevation of arachidonic acid (AA), a precursor for proinflammatory substances that intensify neuroinflammation (violet arrows). Apolipoprotein D (ApoD) can modulate the inflammatory response (1), bind arachidonic acid (2), or directly reduce oxidative stress (3). In PD, ApoD colocalizes inside glial cells next to dopaminergic neurons in the substantia nigra, providing protection against neurotoxicity (4). ApoD: apolipoprotein D; AA: arachidonic acid; GSH: glutathione. Created with https://BioRender.com.

PD is a progressive and neurodegenerative complex disorder that causes symptoms such as tremors, rigidity, bradykinesia (decrement in movement speed and agility), akinesia (difficulty initiating movements), reduced facial expression, small and cramped handwriting, depression, cognitive impairment, and dementia [88,89]. Although PD mainly occurs in the elderly, and most of the patients start showing symptoms at 50 years of age or older, there are some instances of young-onset PD. Before the full development of motor Parkinsonism, there is a prodromal disease in which early signs of PD neurodegeneration are present [90]. As the second most prevalent neurodegenerative disease, PD affects 1% of people over 65 and is the primary cause of Parkinsonism. PD is a growing source of disability and mortality. The prevalence of PD has been projected to double over the next 30 years [54,88,91]. PD is characterized by depigmentation of the substantia nigra and locus coeruleus, accompanied by the loss of neuromelanin-containing dopaminergic neurons in the pars compacta of the substantia nigra, the basal nucleus of Meynert, the dorsal motor nucleus of the vagus nerve, and gliosis in the substantia nigra pars compacta and the pontine locus coeruleus [92,93]. Additionally, there is the presence of Lewy bodies that are eosinophilic intraneuronal cytoplasmic inclusion bodies containing α-synuclein [54,91,94]. PD is a complex disorder with diverse underlying factors; however, the primary etiology of PD remains uncertain. Delving into the molecular theater, mitochondrial dysfunction, α-synuclein misfolding and aggregation, neuroinflammation, abnormalities in protein handling, and oxidative stress are responsible for neuronal loss [54,87,91,94,95]. The imbalance between ROS and antioxidants in the dopaminergic neurons of the substantia nigra contributes to the oxidation of neuronal lipids, proteins, and DNA. It also results in decreased levels of reduced glutathione (GSH), which is associated with an increased release of AA [54,87,95]. GSH is an antioxidant that regulates several cellular events, including gene expression, DNA and protein synthesis, cell proliferation and apoptosis, signal transduction, cytokine production, and protein glutathionylation. GSH deficiency significantly contributes to neuronal oxidative stress [96,97,98]. Since ApoD enhances the antioxidant barrier and stabilizes AA/limits its metabolism, it may be advantageous in the early PD stages (Figure 2) [54,87]. Interestingly, there is an increased ApoD immune signal in glial cells surrounding dopaminergic neurons of the substantia nigra. Still, dopaminergic neurons of the substantia nigra affected by PD do not express ApoD [3,4,50,54,87,95]. All that indicates that elevated levels of ApoD in surrounding glial cells may protect neurons against neurotoxicity. In contrast, nigral dopaminergic neurons are more susceptible to stressors due to their inability to express or assemble ApoD [88]. Therefore, increasing the ApoD level in substantia nigra neurons could offer a promising avenue for treating Parkinson’s disease patients [54,87].

### 3.2. Alzheimer’s Disease

**Figure 3:**
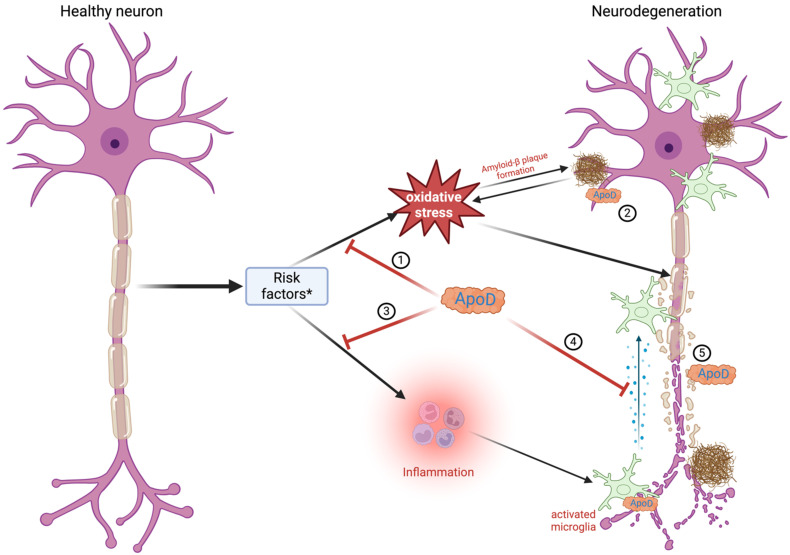
Role of apolipoprotein D in Alzheimer’s disease. Alzheimer’s disease occurs due to multiple risk factors * (age, APOE ε4 gene, genetics, head injury, alcohol, poor diet). These factors result in oxidative stress (1), which leads to the buildup of amyloid-β hindering neuronal activity. ApoD builds up in proximity to amyloid-β plaques, lowering their harmful effects (2). ApoD also inhibits inflammation (3) and decreases the production of proinflammatory cytokines by entering microglial cells (4). ApoD is essential to the regeneration of damaged neurons (5). Created with https://BioRender.com.

According to the WHO, around 47.5 million people suffer from dementia, with 7.7 million new cases every year. AD is the most common cause of dementia, accounting for an estimated 60% to 80% of cases [2,99]. AD is a fatal, progressive, age-related neurodegenerative disorder that manifests symptoms ranging from minor memory lapses to a complete loss of speech, self-awareness, apathy, and immobility. After the age of 65, the incidence and prevalence of AD double every five years. Additionally, people aged 65 and older survive an average of four to eight years following the diagnosis [2]. In AD, characteristic diffuse and mature senile plaques composed of aggregated amyloid-β as well as neurofibrillary tangles (NFTs) formed from hyper-phosphorylated tau protein are observed alongside with synapse and neuronal damage [6,69]. Despite the initial damage in the brain regions that govern memory, language, and cognitive functions, the onset of symptoms is believed to occur 20 years or more after the initial stages of the disease begin [2,69]. The Braak stage measures the severity of the neurofibrillary tangle (NFT) pathology in AD [100]. There are six levels of Braak stages: I and II indicate that NFTs are confined mainly to the entorhinal region of the brain; III and IV indicate the involvement of limbic regions such as the hippocampus; and V and VI indicate moderate-to-severe neocortical involvement. Therefore, patients with higher Braak stages show an increase in cognitive impairment [100]. The fundamental reason underlying AD is the gradual depletion of neurons, mostly in the hippocampus and the cerebral cortex [2]. Oxidative stress represents one of the initial occurrences in AD pathology, possibly before the formation of amyloid-β plaques [6,69]. Additionally, chronic inflammation and alterations in the membrane composition are other pathologies appearing in AD and modulating amyloid-β production [2,6,69]. The antioxidant function of ApoD as a lipid antioxidant may be surpassed by excessive lipid peroxidation, which can damage neuronal membranes, proteins, and mitochondrial structures, further propagating neurodegenerative processes (Figure 3) [71]. ApoD may aggregate and accumulate in a dimeric form within amyloid-β plaques or other insoluble deposits. There is evidence of increased ApoD expression in cortical neurons of sporadic late-onset AD [69,72]. Genetic studies provide further support for the significance of ApoD in the pathophysiology of AD. The *ApoD* gene is localized on chromosome 3q36.2, quite close to the 3q25–26 region linked to AD. Desai et al. [76] have found that polymorphisms in the *ApoD* gene, unique to populations of African American ancestry, could increase the risk of AD. Notably, this occurs only when the *APOE ε4* allele is also present. The *APOE* gene is the most prevalent risk factor of AD and stands for more than half of all cases. Many epidemiological studies indicate that the ε4 allele is associated with amyloid-β deposition and significantly increases the risk of early-age-onset AD [101,102]. Further studies on the Finnish population have shown AD patients to have significant differences in allele and haplotype distributions compared to control subjects [2,4,101,102,103,104]. Helisalmi et al. [72] found that the −352G allele for *ApoD* is associated with an increased risk of early-onset AD, especially in double doses, when found with the nearby +45 C/C *ApoD* genotype and for women. ApoD might be a neuroprotective, antioxidant, and anti-inflammatory molecule, as indicated by its increased presence in the hippocampus, prefrontal cortex, temporal cortex, entorhinal cortex, and neurons/glial cells affected by neurofibrillary tangles, although the number is lower in diffuse plaques as compared to mature ones in AD subjects [3,4,6,76,82,95,105,106]. More studies have found that ApoD colocalizes with amyloid-β within senile plaques of AD-affected brains, probably protecting neurons against amyloid-β-induced cytotoxicity [6,25,69,104,107,108]. Thus, ApoD expression levels may influence the amyloid-β plaque pathology. Moreover, the extent of ApoD overexpression would rely on the glial response following the initial damage and could be implicated in reinnervation processes [6,25,69,104,107,108]. ApoD, as a lipid transporter, may also inhibit the aggregation of amyloid-β or enhance its clearance within the brains of AD individuals [6,25,69,104,107,108]. In 2008, Chen et al. [104] noticed significant interactions between *ApoD* polymorphisms and gender or AD onset age. Several other studies also showed genetic variations associated with higher sporadic AD cases [104]. Hence, ApoD may be important in modulating the risk of sporadic AD [104]. ApoD might additionally influence AD pathology by regulating eicosanoids such as 5s-, 12s-, and 15s-hydroperoxy-eicosatetraenoic acids, which are pivotal in brain inflammatory pathways. Changes in eicosanoid metabolism are expected to profoundly affect neuronal signaling and neuroinflammation in the context of AD [108]. Furthermore, ApoD might also influence the inflammatory pathways linked to AD pathology by regulating certain cytokines. Given that inflammation promotes the production of amyloid-β and worsens the amyloid-β plaque pathology, ApoD may modulate AD progression through anti-inflammatory mechanisms [108].

### 3.3. Multiple Sclerosis

**Figure 4:**
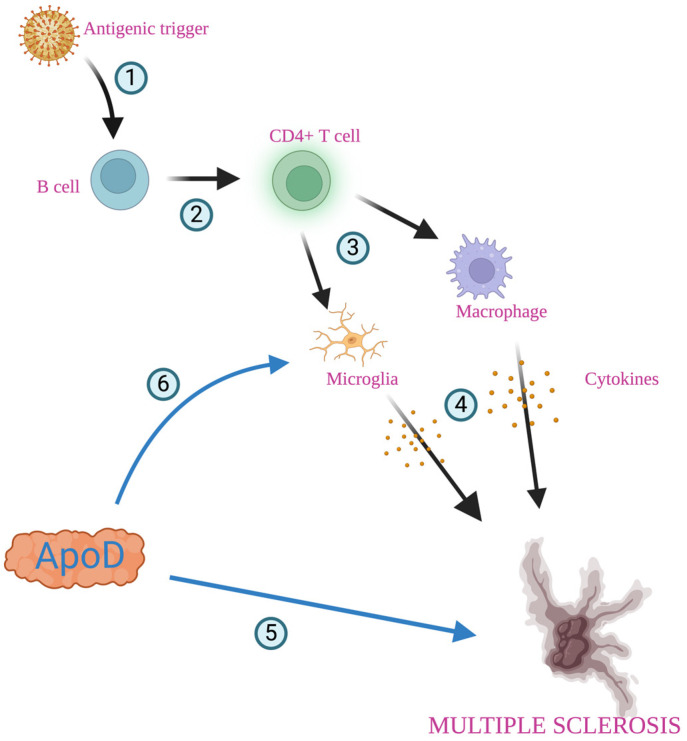
Role of apolipoprotein D in multiple sclerosis. Foreign antigen (most probably Epstein–Barr virus) provides an antigenic trigger through molecular mimicry (1). Antigen-presenting cells, including B cells, may activate CD4+ T cells (2), which interact with microglia and macrophages (3), antibodies, cytokines, and other mediators, and these are released, leading to inflammatory responses and demyelination of neurons (4). ApoD is involved in removing lipids during nerve degeneration and contributes to axonal regeneration and remyelination (5). Inflammation in multiple sclerosis leads to microglia activation (6). Created with https://biorender.com.

Multiple sclerosis (MS) is the most common immune-mediated inflammatory demyelinating disease, affecting over 2.8 million people worldwide [109]. The most common symptoms are numbness, weakness, tremors, loss of vision, pain, and paralysis caused by inflammation, demyelination, and axonal damage [63,110,111]. Based on the clinical representation, there are two major subtypes of MS: relapsing–remitting multiple sclerosis (RRMS) and primary progressive (PP) subtypes [52]. The etiology of MS remains unidentified, but the most likely theory is the immune-mediated disorder characterized by the exacerbated autoreactive lymphocyte reaction against oligodendrocytes/myelin proteins following microglial activation and chronic neurodegeneration [63,110,112,113]. Epstein–Barr virus has been suspected as a possible trigger of MS, providing an antigen through molecular mimicry [114]. Antigen-presenting cells, including B cells, may activate CD4+ T cells. CD4+ cells could induce microglia and macrophages, leading to inflammatory responses and tissue damage [115,116,117]. The characteristic neuropathological feature of MS is the appearance of focal demyelinating lesions in the white matter throughout the brain and spinal cord. Those lesions are accompanied by varying degrees of inflammation and gliosis, with the partial preservation of axons, which can be remyelinated to repair the inflicted damage [63]. Additionally, inflammatory breakdown of the blood–brain barrier has been observed [63]. Patients with MS have abnormally high levels of ApoD in the plasma and CSF [25,31,63,105,106,118,119]. Additionally, increased intrathecal production of ApoD, which correlates with the duration of the disease, has been observed [25,31,63,105,106,118,119]. ApoD is involved in removing lipids during nerve cell degeneration and has been reported to be present in elevated levels in MS (Figure 4) [25,31,63,105,106,118,119]. There are differences in the levels of ApoD within the plaque. In the inactive areas with demyelinated plaques, the levels of ApoD are lower than in the active regions, but they increase again in remyelination areas [63,119]. The absence of ApoD appears to hinder myelin compaction and, more significantly, myelin clearance after injury, leading to a delay in axonal regeneration and remyelination [63]. Inflammation in MS leads to defects in the modulation of the ApoD expression in astrocytes and oligodendrocytes. Together with the death of oligodendrocytes, it may disrupt the demyelination/remyelination balance, making it less effective and contributing to the development of the disease [63].

## 4. Diagnostic Significance of ApoD in Neurodegenerative Disorders

### 4.1. Parkinson’s Disease

According to Heiko Braak’s theory, the pathological changes in PD begin in the medulla oblongata and the olfactory bulb, progressing rostrally over many years to the substantia nigra and midbrain, at which point clinical symptoms and signs are likely to be present, and eventually they affect the cortical regions over time [94]. Dopamine deficiency leads to significant disruptions in connectivity between the thalamus and motor cortex, manifesting as extrapyramidal signs such as bradykinesia and rigidity. However, those symptoms occur when approximately 70% of dopaminergic neurons have degenerated, 50% of dopaminergic neurons in the substantia nigra are lost, and dopamine levels in the striatum are 80–85% depleted, which is why PD is often diagnosed late [94]. However, during the prodromal phase, non-motor symptoms—such as smell loss or constipation—resulting from neurodegenerative changes in extranigral sites are often encountered by patients several years, or even decades, before the appearance of typical motor symptoms in PD. ApoD, associated with the integrity of the dopaminergic system, may serve as a biomarker for identifying patients in the early stages and enabling neuroprotective treatment [88,94]. In 2018, Waldner et al. [54] noticed that there was a significant increase in ApoD in the plasma of patients with PD (mean 104.15 ± 30.96 ng/mL) as compared with age-matched healthy patients (mean 79.35 ± 26.25 ng/mL). ApoD significantly correlates with disease severity, suggesting that ApoD could be a marker for disease progression and effective therapies. Additionally, the ApoD levels in human plasma in PD were found to be unrelated to gender in a group of white Americans [54,94]. Jiang et al. [51] analyzed serum-derived exosomes containing ApoD in 20 patients with mild (*n* = 10) and severe (*n* = 10) PD and healthy controls (*n* = 10). ApoD expression was upregulated in patients with more severe PD compared to mild PD and healthy controls. In contrast, a study by Noguero et al. [120] discovered that patients with severe monoamine neurotransmitter deficiencies, one of the hallmarks of Parkinsonism [121], had lower concentrations of CSF ApoD than those with mild deficiencies. However, symptoms of PD can be similar to those of other neurodegenerative conditions. PD accounts for 3.6% of dementia cases, and 24.5% of people with PD develop dementia, making diagnosis even more challenging [54,87,88,99]. This underscores the importance of finding a biomarker to identify the earliest stages of the disease, assess the risk, and ensure accurate diagnosis. ApoD may be a reliable candidate [54,87,88,99]. However, the limitations of ApoD as a PD biomarker should also be mentioned. To date, many surveys have used relatively small study groups [51,54]. Distinguishing between changes in ApoD levels specific to motor and cognitive symptoms can also be challenging. α-synuclein collected from CSF is established as one of the biomarkers used in PD, but its role in monitoring progression needs to be further validated. In previous studies, immunoassays and standardized antigens for quantification differed between laboratories. In addition, contamination of red blood cells may also limit the usefulness of measuring α-synuclein in CSF [122,123]. Therefore, an advantage of ApoD is the possibility to provide insights into disease-modifying pathways beyond α-synuclein pathology [122].

### 4.2. Alzheimer’s Disease

Amyloid-β42 (Aβ42) is a CSF protein approved as a neurochemical biomarker of AD [9]. Defining a cutoff point that distinguishes between AD and non-AD pathologies with high diagnostic accuracy is challenging [9,124]. Aβ42 also requires invasive measures to obtain lumbar puncture. Therefore, ApoD has shown potential as a less invasive biomarker since it can be detected in plasma and urine [9].

ApoD may serve as a marker for AD due to its upregulation in the hippocampus (by 60–350%) and CSF (by 300%) of AD patients [125,126]. Other studies have noted elevated levels of ApoD in dispersed cortical astrocytes among AD individuals as compared to age-matched controls, although no significant quantitative variances in cortical gray matter regions have been detected [38]. This has been further explored in a clinical study on 90 AD patients with temporal cortex atrophy. The ApoD levels were significantly elevated in the CSF of individuals with middle and inferior temporal cortex atrophy, as confirmed by the MRI scan [53]. Furthermore, ApoD may serve as a glial marker of AD progression since studies on AD patients have shown increased levels of ApoD expression in both neurons and glial cells in the hippocampus, cerebral cortex, and CSF, regardless of gender. Those increases correlate with the Braak degeneration stage, indicating a link to disease progression [2,6,50,99,127]. Lacar et al. [128] analyzed differentially abundant proteins in serum samples from 141 patients with AD and 217 healthy controls. ApoD showed a significant increase in AD patients. The research conducted by Watanabe et al. [55] showed that the ApoD levels in urine measured using the ELISA method were significantly higher than in the control group. In the studies carried out by Terrisse et al. [129], the ApoD concentration in the CSF of AD patients (4.23 ± 1.58 μg/mL, *p* < 0.0001) was 3–4 times higher as compared to the control group (1.15 ± 0.71 μg/mL). The ApoD concentration in the CSF positively correlates with the inheritance of the E4 allele in a dose-dependent manner; however, based on their research, ApoD is not specific to AD. Other neurological pathologies also show elevated levels of ApoD in the CSF [56]. Soares et al. [130] analyzed plasma samples from patients with AD (APOE allele was present in 67.9%), mild cognitive impairment (APOE allele was present in 53.3%), and healthy control (APOE allele was present in 8.6%). There was a decreased level of ApoD in patients with mild cognitive impairment vs. control and a decreased level of ApoD in patients with AD vs. control [130]. Khnedger et al. [56] analyzed plasma levels of circulating extracellular vesicles (cEVs) of ApoD in 15 APOE-ε4-positive individuals with AD and 21 healthy controls with no cognitive impairments. They observed that the plasma cEV levels of ApoD were significantly higher in the AD group than in the controls. In the studies by Oláh et al. [57], the ApoD level was considerably lower in the CSF of AD patients. However, Kuiperij et al. [40] showed no differences in ApoD CSF levels between patients with AD (mean 9.4 ± 5.4 μg/mL) and the controls (mean 7.9 ± 2.9 μg/mL). Rehman et al. [131] reviewed 75 proteins present in CSF, suggesting that ApoD, together with 5 other proteins (apolipoprotein E, α_1_-microglobulin, complement component 3, interleukin 16, insulin-like growth factor binding protein 2), may serve as an effective biomarker to differentiate patients with mild cognitive impairment from AD and cognitively normal individuals. The study by Dauar et al. [132] on 93 participants without cognitive impairment showed that contactin 5, a neuronal membrane protein involved in key neurodevelopmental processes, was positively correlated with CSF ApoD (*p* = 0.000186), which may be due to neuronal damage. In the mouse model, the *ApoD* gene increased at the deafferentation stage, suggesting a role in axonal, terminal, and synaptic remodeling in response to damage to the intraparietal cortex [132]. Future research on genetic polymorphisms within the *ApoD* gene could yield critical insights into how genetic variability impacts its expression and function in neurodegenerative processes. They may also uncover specific polymorphisms associated with altered risk or progression of AD, paving the way for personalized therapeutic approaches. For instance, identifying individuals with specific *ApoD* variants could inform the development of targeted treatments or preventive strategies, such as the pharmacological modulation of ApoD levels or gene therapy. Conflicting findings in ApoD levels in AD may come from small study groups, which may not provide enough statistical power to detect subtle changes. The ApoD level may also exhibit dynamic changes depending on the AD stage. Increased ApoD levels could reflect early compensatory responses to oxidative stress or neuroinflammation, while reduced levels in later stages might indicate a depletion of neuroprotective resources [69,71,72]. Variability in the distribution of APOE genotypes among study cohorts could influence the findings. Therefore, future studies should stratify participants by disease stage, APOE genotype, and co-morbidities to clarify how ApoD levels change throughout AD progression.

### 4.3. Multiple Sclerosis

The ApoD expression increases in MS and may be linked to immune cells and inflammatory response [133]. The research conducted by Reindl et al. [134] showed that the ApoD levels in the CSF were significantly higher in chronic inflammatory demyelinating polyneuropathy and Guillain–Barre syndrome (CIDP/GBS) than in MS and non-inflammatory neurological diseases (NNDs). The findings showed lower serum ApoD levels in MS as compared to CIDP/GBS (*p* < 0.05) and inflammatory neurological diseases (INDs). ApoD indices (ApoD_CSF_/ApoD_serum_:albumin_CSF_/albumin_serum_) are highest in the early MS patients (13.08 ± 5.15) than in relapsing–remitting multiple sclerosis (RRMS) (8.52 ± 2.42) and secondary chronic progressive disease (5.14 ± 3.63). A negative correlation exists between disease MS duration and the CSF ApoD level [134]. Stoop et al. [118] aimed to investigate the identification of proteins in the CSF associated with MS using advanced mass spectrometry techniques. This result was consistent with the findings of the studies conducted by Reindl et al. (127), who showed that ApoD was differentially expressed in the CSF of MS patients compared to the control groups. Further studies by Stoop et al. [52] confirmed that the ApoD levels increased in both primary progressive MS (FC = 2.579) and relapsing–remitting MS (FC = 2.824) as compared to the healthy controls. In 2012, Kroksveen et al. [135] reported decreased levels of ApoD in RRMS patients compared to other neurological diseases and controls, suggesting a potential differential expression in varying disease states. In another study, Kroksveen et al. [136] studied the CSF proteins in the early forms of MS. The ApoD level was significantly less abundant in RRMS patients, who had clinically isolated syndrome at the time of lumbar puncture, as compared to the patients with other inflammatory and non-inflammatory neurological diseases. ApoD levels change during disease development and could indicate disease activity and progression [128]. MS is characterized by both inflammatory (active demyelination) and degenerative (axonal loss) disturbances [137]. Therefore, modulating the function of ApoD can reduce inflammation during relapses and slow the transition to progressive stages of the disease, making it a potential target for future research. To date, there is no serological diagnostic test for MS (biomarkers of true relapses, disease progression, and response to treatment); therefore, the use of ApoD may be beneficial in MS [10].

## 5. Conclusions and Future Prospects

**Figure 5:**
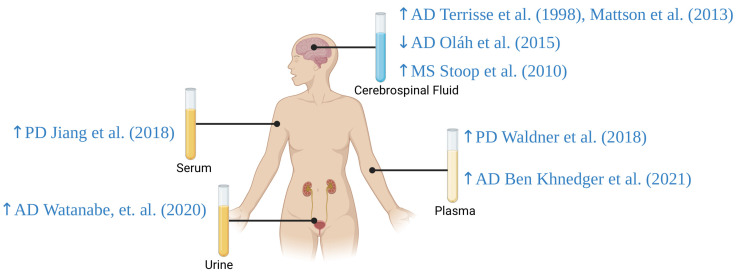
ApoD levels in plasma, serum, cerebrospinal fluid, and urine in patients with selected neurodegenerative diseases. AD: Alzheimer’s disease; MS: multiple sclerosis; PD: Parkinson’s disease; ↑: increased Apo D levels; ↓: decreased Apo D levels [51,52,54,55,56,57,129]. Created with https://biorender.com.

AD, PD, and MS account for significant global health challenges due to their prevalence, progressive nature, and impact on the quality of life and economic burden. NDs share common causative factors, including genetic predispositions, environmental stressors, and complex molecular mechanisms such as oxidative stress, disturbances in lipid metabolism, and chronic inflammation. Identifying reliable biomarkers of NDs is critical for early diagnosis, disease progression monitoring, and therapeutic responses. ApoD has emerged as a promising candidate in this regard. ApoD, a small glycoprotein involved in lipid metabolism and oxidative stress regulation, is abundantly expressed in the CNS and various body fluids. ApoD expression levels and molecular forms differ across tissues, which could provide valuable insights into the state of the blood–brain barrier and neurodegenerative conditions. ApoD plays a protective role in CNS diseases by modulating inflammatory responses, aiding lipid transport, and mitigating oxidative stress. Elevated levels of ApoD in the brain and body fluids have been associated with neuroprotective effects, such as reduced lipid peroxidation and enhanced neuronal survival. In PD, increased ApoD levels in glial cells surrounding affected dopaminergic neurons suggest a protective response against neurotoxicity. In AD, ApoD colocalizes with amyloid-β plaques and may mitigate amyloid-β-induced cytotoxicity and inflammation. Additionally, the modulation of inflammatory pathways and lipid metabolism by ApoD underscores its role in the pathophysiology of NDs. Future research should focus on developing non-invasive diagnostic tools for detecting ApoD in body fluids, which could facilitate the early diagnosis and monitoring of NDs (Table 1, Figure 5). Exploring the therapeutic potential of ApoD could pave the way for new treatments aimed at slowing down disease progression and improving patient outcomes. The use of gene therapy to increase ApoD expression in specific brain regions may enhance its protective effects. Pharmacological strategies may aim to increase ApoD synthesis or stabilize its active form to counteract oxidative damage in NDs. Research may also focus on modulating lipid transport. Although there are only a few clinical studies of ApoD, its biological functions suggest that ApoD may become a key component of future diagnostic and therapeutic strategies in NDs. Given the complexity and diversity of NDs, a multifaceted approach that includes biomarkers like ApoD will be crucial for advancing our understanding and managing those debilitating conditions. There is a need for standardized testing protocols to ensure the reliable measurement of ApoD levels across diverse populations. Further studies are required to validate its potential as a diagnostic biomarker; it is necessary to perform larger, well-characterized population studies. It is unclear whether the findings are generalizable to diverse ethnic groups or populations with different genetic risk factors. Studies measuring ApoD in different body fluids require standardized sample collection and analysis protocols since it may yield different outcomes due to the unique biological environments. Furthermore, understanding the genetic regulation of ApoD may also aid in elucidating its broader role in lipid metabolism and oxidative stress. ApoD should also be evaluated along with other markers to improve its specificity.

## Figures and Tables

**Table 1:** ApoD levels in selected neurodegenerative diseases.

Disease	Diagnostic Material	Subjects (Mean Age in Years)	Outcome	*p*-Value	Reference
PD	Plasma	66 patients with PD (72.84 ± 7.07) 19 age-matched healthy controls (72.79 ± 1.55)	↑ ApoD in PD (mean 104.15 ± 30.96 ng/mL) patients compared to controls (mean 79.35 ± 26.25 ng/mL)	*p* < 0.005	Waldner et al. (2018) [54]
	Serum	20 patients (78): 10 with mild PD and 10 with severe PD 10 healthy controls (79)	↑ ApoD in patients with severe PD in comparison to the mild PD and healthy group	*p* < 0.05	Jiang et al. (2019) [51]
AD	CSF	41 patients with AD (75.4 ± 9) 11 patients without neurological diseases (67.4 ± 11)	↑ ApoD in AD (4.23 ± 1.5 8 μg/mL) patients compared to controls (1.15 ± 0.71 μg/mL)	*p* < 0.0001	Terrisse et al. (1998) [129]
			ApoD ↑ in ε4/ε4 AD (5.8 ± 0.73 µg/mL) patients compared to ε3/ε4 AD patients (4.78 ± 0.6 µg/mL)	*p* = 0.005	
			ApoD ↑ in ε3/ε4 AD patients compared to ε3/ε3 AD patients (3.05 ± 0.19 µg/mL)	*p* = 0.001	
	CSF	25 patients with AD (72.04 ± 5.03) 25 patients without neurological diseases (74.52 ± 2.48)	↓ ApoD AD in patients compared to controls (0.62 ± 0.17) ^a^	*p* ≤ 0.001	Oláh et al. (2015) [57]
	CSF	27 patients with AD (69.0 ± 8.4) 67 age- and sex-matched healthy controls (64.8 ± 9.9)	No differences between patients with AD (mean 9.4 ± 5.4 μg/mL) and controls (mean 7.9 ± 2.9 μg/mL)	*p* = 0.1008	Kuiperij et al. (2020) [40]
	CSF	90 patients (44 females, 22 APOE ε4 carriers, mean age 76 years [range 62–90])	↑ ApoD in patients with middle temporal cortex atrophy	*p* < 0.01	Mattson et al. (2014) [53]
			↑ ApoD in patients with inferior temporal cortex atrophy	*p* < 0.05	
	Plasma cEVs ApoD in plasma	15 CIND-AD ^b^ patients (81.4 ± 4.5) 21 controls with no cognitive impairments (79.0 ± 5.2)	No correlation between ApoD in plasma and controls	-	Ben Khnedger et al. (2021) [56]
			cEVs APOD↑ in APOE ε4-positive CIND-AD patients compared to control	*p* < 0.01	
			cEVs ApoD does not correlate between APOE ε4-negative CIND-AD patients and healthy controls	-	
	Urine	18 patients with AD (72.9 ± 5.6) 18 healthy controls (72.8 ± 5.2)	↑ApoD in AD patients compared to control	*p* = 0.003	Watanabe, et. al. (2020) [55]
MS	CSF	11 patients with RRMS ^c^ (43.9 ± 14.1), 10 patients with PPMS ^d^ (48.1 ± 9.0), 10 healthy controls (51.1 ± 13.7)	2.82-fold ↑ ApoD in RRMS patients compared to controls	*p* = 0.0051	Stoop et al. (2010) [52]
			2.58-fold ↑ ApoD in PPMS patients compared to controls	*p* = 0.0054	

^a^ Ratio compared to controls ± standard error of the mean (S.E.M), ^b^ cognitively impaired individuals without dementia converted to AD, ^c^ relapsing–remitting multiple sclerosis, ^d^ primary progressive multiple sclerosis. All statistically significant results (*p* < 0.05) are written in bold. ↑: increased Apo D levels; ↓: decreased Apo D levels. AD: Alzheimer’s disease; CSF: cerebrospinal fluid; MS: multiple sclerosis; PD: Parkinson’s disease.
